# A Case of Sunstroke

**Published:** 1889-12

**Authors:** E. W. Berridge

**Affiliations:** London


### A CASE OF SUNSTROKE.

E. W. Berridge, M. D., London.

Mrs. W. aged fifty, sent for me on May 23d, 1889. Two days previously had gone out-of-doors at seven P. M., and hurried very much, the weather being extremely hot. She returned home at eight P. M., and at nine P. M. was seized with head symptoms, which have continued ever since. Thinking it was "biliousness" she took *Nux* and *Pulsat.* in low potency, but without relief. (So much for domestic Homœopathy and amateur prescribing.) I saw her about 9.30 A. M. She complained that everything seemed jumping, worse when sitting up or from talking for a long time, or if she closes eyes. Fears she will lose consciousness. Face very red, and she feels hot. Yesterday vomited some tea, which was "as bitter as gall." Her brother saw her to-day, and said it was like an attack of sunstroke, such as he had often seen in India, and that she ought to have sent for me sooner.

At ten A. M. I gave her *one dose* of *Thuja*$^{cm}$ (F. C.).

May 24th, four P. M.—Improved last evening, and is now sitting up in another room. This morning the "jumping" had gone, but left a tight feeling all over head, like the sensation of a tight glove on the hand, with a full feeling in the internal ears; this is now better since a sleep of an hour, from which she has just awakened. Face less flushed, and less fever.

May 25th.—No symptoms except weakness.

May 27th.—Well.

Three years ago had a similar attack, and a second one subsequently. On each occasion had no treatment, but had to stay in bed three or four days, and some days elapsed subsequently before she was well. These first two attacks she attributes to worry. This attack had passed off much sooner than before, showing the superiority of Homœopathy to the unaided efforts of nature.

This case shows the curative power of the single dose of the highest potency, even in a grave and acute disease, *where it is the simillimum.*

The remedy was *Thuja*, one of the most frequently indicated remedies in that form of dyscrasia called by Hahnemann *sycosis*, and by Grauvogl *the hydrogenoid constitution.* A frequent symptom in these cases is that the patient is worse in damp weather, or from baths. Hence it is necessary in such cases to preclude the external use of water, *except for purposes of cleanliness.* But whether cleanliness itself must be ignored in these cases is a question on which the opinion of *experienced* and *thoughtful* colleagues would be desirable.

In a contemporary journal a colleague writes that he gives all his patients a printed notice enjoining that they should not resort to even "ordinary sponge baths used for the purpose of cleanliness" without his august sanction; adding that "if patients cannot or will not adopt the above simple but necessary rules whilst under treatment, they had better not begin," as he "does not pretend to work miracles, or do that which is impossible." (Now, Doctor, who on "airth" ever supposed you could work a miracle?) Accordingly, in the case he quotes, he "stopped all meddling with water except on uncovered parts." It is not surprising that the patient, "aged twenty-four, with golden auburn hair, dark hazel eyes, and a lovely fair complexion, five feet in height, and very handsome," felt considerable "chagrin and disappointment." The beauty of the fair patient seems to have made considerable impression on the Doctor's susceptible organism; and, while congratulating him on his good fortune, I wonder that he had the heart to forbid her to use such means as would preserve the delicate whiteness of her skin, which I feel sure must have been one of her characteristics, though he has unaccountably omitted to mention it.

But the question is, *is it necessary to forbid ordinary cleanliness of the entire body?* Cleanliness is one thing, and excessive ablutions another. I, for one, cannot indorse this new THERAPEUTICS OF DIRT.

